# Epithelial cells mimic immune cells: a novel path toward tumor immunotherapy

**DOI:** 10.20892/j.issn.2095-3941.2020.0406

**Published:** 2021-07-09

**Authors:** Ruining Gong, Yan Huang, Xiaoxuan Wang, Xiaobing Chen, Zibin Tian, He Ren

**Affiliations:** 1 Department of Gastroenterology, Center of Tumor Immunology and Cytotherapy, Medical Research Center of The Affiliated Hospital of Qingdao University, Qingdao 266003, China; 2 Department of Human Resources, The Affiliated Hospital of Qingdao University, Qingdao 266003, China; 3 Department of Oncology, The Affiliated Cancer Hospital of Zhengzhou University, Henan Cancer Hospital, Zhengzhou 450008, China; 4 Department of Gastroenterology, The Affiliated Hospital of Qingdao University, Qingdao 266003, China

Recently, we have shown that FOXP3, a critical transcription factor in regulatory T cells (Tregs), is expressed in pancreatic epithelial cells, and restrain the activity of CD8+ T cells by upregulating PD-L1, which in turn regulates immune escape in pancreatic ductal adenocarcinoma (PDAC)^1^. On the basis of a series of studies of our laboratory, we hypothesize that a subset of pancreatic epithelial cells mimics the phenotype and function of Tregs (named quasi-Tregs or qTregs); this concept has been supported by a peer-reviewed commentary^2^. Moreover, evidence suggests that tumor epithelial cells can mimic other types of immune cells and participate in the formation of a tumor immunosuppressive microenvironment (TIM).

The typical pathological features of PDAC include uncontrollable inflammation, which contributes to fibrosis and hypoxia. Hypoxia-inducible factor 1 (HIF-1) is a transcription factor that is activated in hypoxic environments and subsequently sustains tumor cell proliferation and activity. HIF-1 also regulates FOXO1, a key transcriptional factor for expression of FOXP3, thereby regulating FOXP3 expression in tumor tissues^3,4^. Lang’s study^3^ provides novel information on the mechanism of FOXP3 expression in PDAC epithelial cells and suggests that the HIF-1/FOXP3 pathway may be a potential therapeutic target for PDAC treatment.

In the tumor microenvironment, FOXP3+ Tregs inhibit CD8^+^ T lymphocytes and consequently facilitate tumor growth. Moreover, PD-L1 overexpression in PDAC tissues and cell lines is closely associated with cancer-FOXP3 (C-FOXP3)^1^, which inhibits the activity of CD8^+^ T cells by upregulating PD-L1, which in turn modulates immune escape in PDAC. This finding further highlights the importance of C-FOXP3 in generating a TIM for PDAC. Furthermore, C-FOXP3 is expressed in pancreatic cancer cells and acts synergistically with C-C motif chemokine ligand 5 (CCL5), thereby contributing to immune evasion^5^. Interestingly, FOXP3^+^ epithelial cells promote the infiltration of Tregs in pancreatic lesions by directly regulating the CCL5 transcript, and anti-CCL5 antibodies can achieve good antitumor efficacy in C-FOXP3 overexpressed tissues. Furthermore, tumor growth is inhibited and overall survival is more significantly improved in mice overexpressing C-FOXP3 after co-administration of anti-PD-L1 and anti-CCL5 antibodies, as compared with monotherapy. This study suggests favorable combination strategies with curative potential for PDAC. Inhibiting or impairing the functions of qTregs may serve as an attractive approach to cancer immunotherapy.

Interleukin 35 (IL35) is a potent immunosuppressive molecule secreted by Tregs. We have provided evidence that IL35 recruits infiltrating monocytes by regulating the expression and secretion of CCL-5, and that the secretion of CXCL-1/CXCL-8 by monocytes is pro-angiogenic^6^. We have uncovered the mechanism underlying VEGF-independent angiogenesis, which may potentially enable a new clinical treatment modality. EHF deficiency in tumor epithelial cells negatively regulates E-cadherin, thus enabling tumor cells to acquire high metastatic potential *via* epidermal-mesenchymal transformation^7^. EHF methylation of pancreatic epithelial cells promotes release of TGF-β, which in turn induces Treg conversion^8^. Moreover, EHF deficiency promotes the release of GM-CSF and the accumulation of MDSCs in the pancreas. These above-mentioned studies suggest that EHF loss promotes epithelial-mesenchymal transition of malignant epithelial cells that mimic the morphological and phenotypic characteristics of Tregs and are also involved in TIM formation.

We have demonstrated that qTregs are correlated with malignant transformation, immune escape, and the presence of highly invasive epithelial cells induced by uncontrolled inflammation; our findings highlight how epithelial cells mimic Tregs. This study extends TIM research and further expands the traditional views on the roles of tissue-infiltrating immune cells. It also reveals the physiological functions of immunomodulatory molecules and provides new insights into targeting tumor angiogenesis and metastasis. Furthermore, immunoregulatory signals can be used by PDAC epithelial cells, and RORγ, a regulator of inflammation and T cell differentiation, is up-regulated in PDAC^9^.

Similar findings have been validated in other tumors. Liu et al.^10,11^ have found that the lymphocyte-restricted transcription factors Aiolos and SpiB are ectopically expressed in lung cancer cells. This discovery explains why small cell lung cancer has morphological similarities to lymphoma, and provides an example of lymphocyte mimicry in epithelial cancer metastasis, thus providing new insights into tumorigenesis. With the development of single-cell RNA sequencing, various cell subpopulations have been uncovered. In nasopharyngeal carcinoma samples, Jin and colleagues^12^ have found an epithelial-immune dual feature of malignant cells, which express not only classic epithelial markers but also several immune markers. This dual feature is closely associated with poor clinical prognosis and enhanced tumorigenesis. Moreover, this cell subset has stronger immunosuppressive and regulatory functions on T cells in the immune microenvironment. Ultimately, these findings support the presence of phenotypic changes in the epithelium.

The proposal that epithelial cells express the main regulatory factor found in lymphocytes and also mimic their function is notable because it suggests that tumor epithelial cells may be a promising target for immunotherapeutic options. However, this concept raises several questions that remain to be answered. The cell subset originates from mesenchymal cells or epithelial cells, and its role and mechanism in tumor development must be further confirmed. The specific mechanism of the subset involved in the formation of the immune microenvironment and immune escape also remains to be elucidated. Furthermore, comprehensive studies exploring origin and complex immune mechanisms remain necessary.
